# Variance Risk Identification and Treatment of Clinical Pathway by Integrated Bayesian Network and Association Rules Mining

**DOI:** 10.3390/e21121191

**Published:** 2019-12-04

**Authors:** Gang Du, Yinan Shi, Aijun Liu, Taoning Liu

**Affiliations:** 1 School of Business and Administration, Faculty of Economics and Management, East China Normal University, Shanghai 200062, China; gdu@dbm.ecnu.edu.cn (G.D.); nusdugang@foxmail.com (Y.S.); 2 Department of Management Engineering, School of Economics & Management, Xidian University, Xi’an 710071, China; tnliu@stu.xidian.edu.cn

**Keywords:** clinical pathways, data mining, risk factors, variance treatment

## Abstract

With the continuous development of data mining techniques in the medical field, variance analysis in clinical pathways based on data mining approaches have attracted increasing attention from scholars and decision makers. However, studies on variance analysis and treatment of specific kinds of disease are still relatively scarce. In order to reduce the hazard of postpartum hemorrhage after cesarean section, we conducted a detailed analysis on the relevant risk factors and treatment mechanisms, adopting the integrated Bayesian network and association rule mining approaches. By proposing a Bayesian network model based on regression analysis, we calculated the probability of risk factors determining the key factors that result in postpartum hemorrhage after cesarean section. In addition, we mined a few association rules regarding the treatment of postpartum hemorrhage on the basis of different clinical features. We divided the risk factors into primary and secondary risk factors by realizing the classification of different causes of postpartum hemorrhage after cesarean section and sorted the posterior probability to obtain the key factors in the primary and secondary risk factors: uterine atony and prolonged labor. The rules of clinical features associated with the management of postpartum hemorrhage during cesarean section were obtained. Finally, related strategies were proposed for improving medical service quality and enhancing the rescue efficiency of clinical pathways in China.

## 1. Introduction

The Ministry of Health of the People’s Republic of China (MOH) issued documents in January 2010 to formally implement clinical pathways and set up a corresponding standard system. As a standardized medical quality management system, a clinical pathway is a process of diagnosis and treatment for specific diseases according to the three principles of advanced arrangement, independent setting, and the PDCA (plan–do–check–act) cycle [1,2], which plays an important role in shortening the process of diagnosis and treatment, regulating medical behaviors, ensuring medical safety, reducing treatment and service costs, and improving medical service quality and patient satisfaction [3,4].

In recent years, studies on the establishment and standardization of clinical pathway management are increasing. With the development of data mining techniques in the medical field, more scholars have begun to use various data mining algorithms to provide new approaches for the establishment of clinical pathways. Fundamentally, it is essential to identify what medical behaviors are indispensable and the amount of time each of these medical behaviors require [5,6]. In order to provide accurate quantitative timing information for critical medical behaviors, Huang et al. [7,8] developed a new approach of clinical pathway process mining that analyzed data by querying sets of clinical pathway information based on specific clinical workflow behavior logs. This method not only succeeded in finding the order in which key medical behaviors are implemented, but also provided a quantified time series for each behavior. Tsumoto et al. [9] proposed a clinical pathway construction method based on double clustering (attribute clustering and sample clustering) analysis, of which the actual operating results were similar to those of medical experts who manually recorded the clinical pathway.

Clinical pathway variance is a significant problem to be solved [10]. Some scholars have focused on clinical pathway variance analysis. Clinical pathway variance can be classified according to the source of variance [11], and the key problems related to clinical pathway variance analysis have been summarized [12]. Ye et al. proposed a framework for semantics-based clinical pathway and variance management to enable the computerized implementation of clinical pathways [13], and proposed a hybrid exception handling approach based on a generalized fuzzy event–condition–action rule and a typed fuzzy Petri net extended by process knowledge [14]. Additionally, Ye et al. [15] proposed an ontology-based approach of modeling clinical pathway workflows. The action research framework for clinical pathways was proposed, which was progressively modified to suit clinical environments [16]. Significant predictive factors of clinical pathway variance include complications and an abnormal body mass index [17]. The growing use of clinical pathways by nurses and midwives was studied in [18], and the authors concluded that clinical pathways may have significant impacts on nursing and midwifery as professions.

We conducted a model to deal with variance monitoring and handling for clinical pathway management [19], and a knowledge extraction algorithm and a hybrid learning algorithm (for variance) were proposed [20,21]. Niemeijer et al. [22] used the Six Sigma technique to strengthen the variance analysis of elderly hip-fracture clinical pathways. Specifically, the authors designed a retrospective, prospective and non-randomized controlled trial to identify and intervene variances that lead to prolonged hospitalization in elderly patients with hip fractures.

To construct a reasonable Bayesian network for predicting cesarean section clinical pathway variance, immediate causes of postpartum hemorrhage need to be analyzed. Scholars have adopted a retrospective analysis method by which to investigate and summarize the risk factors resulting in postpartum hemorrhage in cesarean section, and concluded that uterine inertia, placental related factors, birth canal injury, and coagulopathy are the main risk factors [23,24]. Relevant experts also agree with this opinion, and hence the above four reasons are taken as parent nodes in the Bayesian network.

By studying and summarizing previous researches, we found that most of the studies on clinical pathway variance were organized by retrospective investigation and used the regression analysis method. Some literature has put forward methods of data mining and mechanisms of variance handling. For the Bayesian network, however, the design in the prediction of clinical path variance is fuzzy. This paper collects medical data regarding cesarean section clinical pathways from the Maternal and Child Health Hospital in Shanghai and performs data preprocessing. Ultimately, an integrated data mining method was used to analyze the variance of postpartum hemorrhage in the cesarean section clinical pathway.

The remainder of the article is organized as follows. In Section 2, the specific methods are presented. The results and outcomes are provided in detail in Section 3. In Section 4, some discussions are mentioned. Finally, Section 5 provides the conclusions and directions for future research.

## 2. Methods

### 2.1. Data Preprocessing

In this paper, the data of 200 cases of diagnosis and treatment about clinical pathways of cesarean section were collected from the Maternal and Child Health Hospital in Shanghai from 2015 to 2016. SPSS (Statistical Product and Service Solutions) as used for data preprocessing to compensate for the defects in the collected data. Adopting the logistic regression analysis method using Stata software, it was found that the following variables were the main risk factors for postpartum hemorrhage after cesarean section: prolonged labor, multifetation, effect of oxytocin, macrosomia, abnormal fetal position, complications of pregnancy, pregnancy associated with coagulopathy, placenta implantation, placenta praevia, placental abruption, and placental adhesion.

### 2.2. Integrated Risk Factor Identification and Variance Handling of Clinical Pathways

The methodology of integrated risk factor identification and variance handling of clinical pathways can be divided into two parts. One is risk factor identification based on the Bayesian network, and the other is variance handling of clinical pathways using association rule mining.

**Figure 1:**
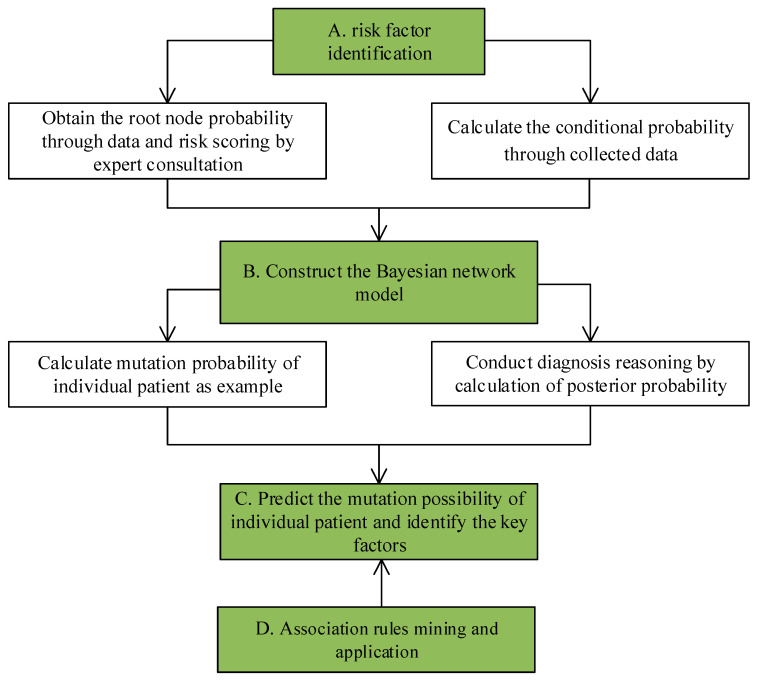
The flow chart of integrated risk factor identification and variance handling of clinical pathways.

According to [25], the analysis flow chart of the integrated risk factor identification and variance handling of clinical pathways is shown in Figure 1.

#### 2.2.1. The Theoretical Framework of the Bayesian Network Model

Comprehensively considering the risk factors identified through the above regression analysis, gynecological advice from the Shanghai International Peace Maternal and Child Health Care Hospital, and consultation of the relevant literature [25], the risk factors can be divided into two categories: primary risk factors and secondary risk factors, respectively. Through identification of the risk factors, we built the Bayesian network structure and determined the prior probability of each root node and the conditional probability table of each child node.

#### 2.2.2. Analyzing the Critical Risk Factors by Diagnostic Reasoning

A Naive Bayesian algorithm is a commonly used classifier that assumes that the influence of an attribute value on a given class is independent of the values of other attributes [26,27]. For the input of the sample set, we first calculated the joint probability distribution between the input and output, according to the conditional independence condition of the future. We then input samples of X to calculate the maximum posterior probability distribution of Y. We gave a sample (x, y), and defined X tuples as a vector set of n dimensions (X = {x_1, x_2, …, x_n}, and similarly Y = {y_1, y_2, …, y_k}). Then we defined P(X, Y) as the joint probability distribution of both. The naive Bayesian classifier on the basis of the feature attribute values were conditionally independent on each other (that is, there was no dependency between attributes), so the conditional probability distribution of x based on y can be written as follows:

(1) $$P\left(X=x|Y=y_{k}\right)=P\left(X_{1}=x_{1},\ldots ,X_{n}=x_{n}|Y=y_{k}\right)\;=\prod_{r=1}^{n}P\left(X_{r}=x_{r}|Y=y_{k}\right)$$

Naive Bayesian is based on the assumption of feature independence to obtain the joint probability distribution of X and Y. Most features are actually a relationship, and the independence of features makes the naive Bayesian model relatively simple and the efficiency greatly improved; for this reason, the Naive Bayes classification method is also called the simple Bayesian classification method.

When we need the simple Bayesian to classify the number of x, we need to calculate the posterior probability $\;P\left(Y=y_{k}|X=x\right)$, which according to Bayes’ theorem can be obtained by

(2) $$P\left(Y=y_{k}|X=x\right)=\frac{P\left(X=x|Y=y_{k}\right)\;P\left(Y=y_{k}\right)}{\sum_{k}P\left(X=x|Y=y_{k}\right)\;P\left(Y=y_{k}\right)}$$

The conditional probability distribution of Y based on X according to the conditional independent hypothesis is obtained.

After calculating the probability of all the above nodes, the key risk factors can be found by backward reasoning. One of the backward reasoning methods of the Bayesian network is diagnostic reasoning. The specific approach is to assume the occurrence of postpartum hemorrhage after cesarean section, and to find out the cause of the result by backward reasoning. The principle is that the posterior probability is calculated to obtain the specific probability distribution of the parent node of each child node; thus, the most important factor affecting the child nodes can be known by sorting the probability distribution of the parent node. The posterior probability formula for calculating the Bayesian network is as follows:

(3) $$P\left(Y_{m}|X\right)=\frac{P\left(Y_{m}\right)P\left(X|Y_{m}\right)}{\sum P\left(Y_{i}\right)P\left(X|Y_{i}\right)}(i=0,1,2\ldots m\ldots n)$$

where $P\left(Y_{m}|X\right)$ in the formula refers to the posterior probability of $Y_{m}$ when event X occurs in the state; $P(Y_{m})$ is the prior probability of different events $Y_{i}$; and $P\left(X|Y_{i}\right)$ is the conditional probability of the occurrence of event *X* when different events $Y_{i}$ occur. The larger the value of $P\left(Y_{m}|X\right)$ is, the higher its posterior probability is, and the greater affect $Y_{m}$ would have on its child node X. In this way, we can find the primary risk factors and secondary risk factors leading to postpartum hemorrhage after cesarean section.

#### 2.2.3. Association Rule Mining and Application

By continuing to search for the maximum frequent item and pruning based on minimum support, the Apriori algorithm can constantly generate different association rules from frequent item sets. Support and confidence are measures of the strength of association rules. Support is to determine the proportion of association rules of a given data set. The minimum support provides a minimum standard for the proportion of the destination items of the overall data set [28]. On the one hand, the proposed risk factor identification method for clinical pathway variance based on the Bayesian network combines qualitative and quantitative methods so that it can obtain more accurate and reliable computational results. On the other hand, the proposed treatment method for clinical pathway variance based on association rule mining can generate dominant clinical decision support rules, and be more easily understood and applied by clinical decision makers when compared with other data mining methods such as neural networks and the support vector machine.

## 3. Results

### 3.1. Risk Factor Analysis on Cesarean Section Variance

#### 3.1.1. Construction of the Bayesian Network Model

The primary and secondary risk factors are generated based on the above regression analysis, in which the primary risk factors have a direct impact on clinical pathway variance, and the 11 secondary risk factors have an influence on the primary risk factors. For example, a prolonged labor may cause uterine atony, followed by postpartum hemorrhage after cesarean section. The name of the specific factors and relationships between them are shown in Table 1.

**Figure 2:**
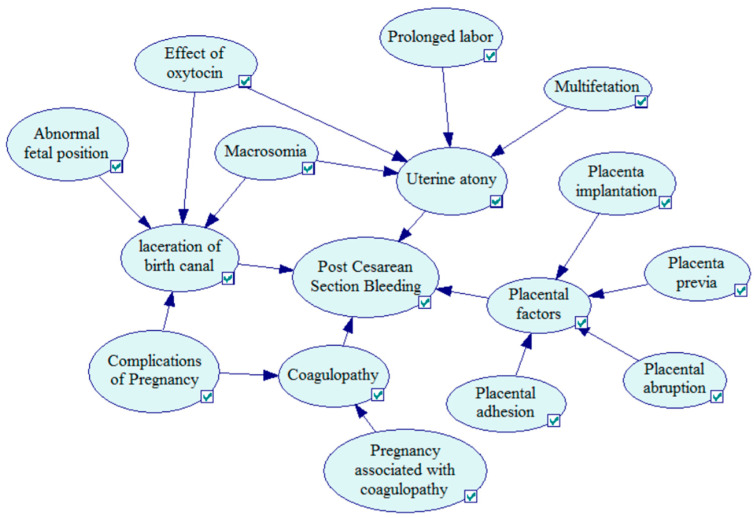
The Bayesian network structure for postpartum hemorrhage after cesarean section.

The risk factor system will be transformed into the parent node and the child node in the Bayesian network structure. The secondary risk factors are the child node of the primary risk factors. The primary risk factors are the child node of the clinical pathway variance result. Through the identification of the risk factors in Table 1, we built the Bayesian network structure for postpartum hemorrhage after cesarean section using GeNIe2.0 software (software version 2018.1-4.0.784.31), which is shown in Figure 2. The symbolic representation of nodes is shown in Table 2.

Next, we need to define the range of each variable. The range of *A* is (State 0, State 1). If the case of postpartum hemorrhage after cesarean section occurs, it would be State 1; otherwise, it would be State 0. The range of $B_{i}(i=1,2,3,4)$ and $C_{m}(m=1,2,3\cdots 11)$ are also (State 0, State 1). State 0 indicates that the factor did not occur and had no impact on its child nodes, and the definition of State 1 is the opposite.

In addition to constructing the network structure, the Bayesian network model needs to determine the prior probability of each root node and the conditional probability table with each child node. Combining the feedback data and questionnaires, the value for the root node probability $P\left(C_{i}\right)\left(i=1,2\cdots 11\right)$ is determined by the occurrence degree $R\left(C_{i}\right)\left(i=1,2\cdots 11\right)$ of the risk factors given by experts and the possibility of the risk $P_{d}\left(C_{i}\right)\left(i=1,2\cdots 11\right)$ obtained by data analysis. The probability formula is as follows:

(4) $$p\left(c_{i}\right)=R\left(c_{i}\right)\times P_{d}\left(c_{i}\right)\;\left(i=1,2\cdots 11\right)$$

Risk would be higher if two multipliers had larger values. Therefore, it is significant to determine the marginal probability of each risk factor such as the probability of uterine atony of the parent node probability (Table 3).

As the conditional probability does not need to be revised based on subjective opinions, the objective reality should be maintained. Therefore, the conditional probability table of each node can be established by gathering the data for clinical pathway implementation. Table 4 shows a conditional probability for coagulopathy.

It is obvious from Table 4 that when the two parent nodes *C*_6_ (complications of pregnancy) and *C*_11_ (pregnancy associated with coagulopathy) are in different states, there is a probability of *B*_4_ (coagulopathy) occurrence. For example, when *C*_6_ and *C*_11_ occur at the same time, both of them are under the condition of State 1, and the probability of *B*_4_ occurrence in a gravida’s physiological manifestation is 0.78.

After acquiring the prior probability of the root node of the Bayesian network structure (secondary risk factors) and the conditional probability of all child nodes, the occurrence probability of each child node can be further calculated. The primary and secondary risk factors are independent from each other and the probability of a child node is a combination of each irrelevant risk factor.

**Figure 3:**
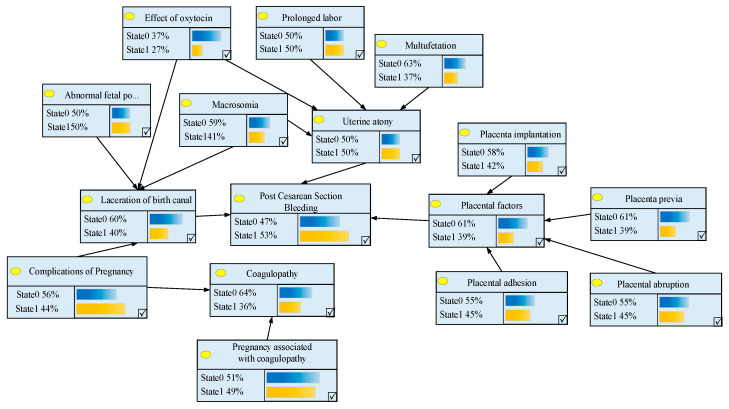
The risk calculation results of postpartum hemorrhage using the Bayesian network.

We can obtain the probability when the value of the node *B*_4_ (coagulopathy) is State 1. Therefore, the probability of pregnancy having coagulopathy is 0.3636. The probability of other nodes can be similarly obtained, such as the probability of postpartum hemorrhage after cesarean section (53%). The results of the Bayesian network model are presented in Figure 3.

#### 3.1.2. The Risk Prediction of Postpartum Hemorrhage Variance

We can predict the probability of postpartum hemorrhage using the above Bayesian network prediction model. In this study, a case was used to verify the feasibility of the risk prediction of postpartum hemorrhage for individual patients. When gravidas are detected with gestation cru or functional disorder and individual clinical problems of placenta implantation and other placenta symptoms in prenatal diagnosis, that is to say, it is known that $P\left(C_{1},C_{4},C_{7},C_{8},C_{11}=State1\right)=1$, the exact probability of the patient’s postpartum hemorrhage condition P(A=State1) can be calculated by using the full probability formula mentioned in the previous section.

The difference of calculating the probability in the premise of the assumption can be found through the process of calculating the cru or dysfunction as State 1. Thus, it can be seen that if a pregnant woman has gestation cru or functional disorder, the probability of coagulopathy is 0.49. By the same method, the probability of the primary risk factors, which have been affected by individual clinical features of gravidas, can be obtained in turn:

$$\begin{array}{l} P\left(B_{1}=State1\right)=0.72,\\ P\left(B_{2}=State1\right)=0.48,\\ P\left(B_{3}=State1\right)=0.59. \end{array}$$

**Figure 4:**
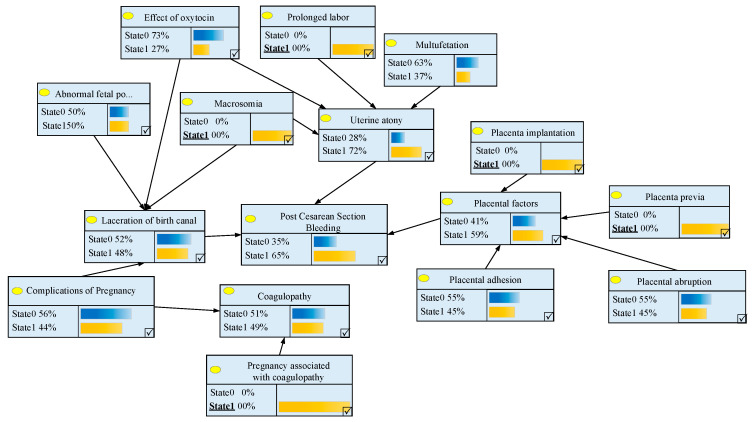
The risk of postpartum hemorrhage for individual pregnant woman.

Through the probability of the primary risk factors, we can deduce that the probability that the gravidas would have a sudden postpartum hemorrhage condition is P(*A* = State 1) = 0.65. The probability of each node in the Bayesian network is given in Figure 4.

#### 3.1.3. Analyzing the Critical Risk Factors by Diagnostic Reasoning

In order to acquire the posterior probability of $C_{11}$ (gestation cru or functional disorder), we can assume that coagulopathy has occurred; that is, $B_{4}=State1$. The posterior probability can then be solved using the method above.

**Figure 5:**
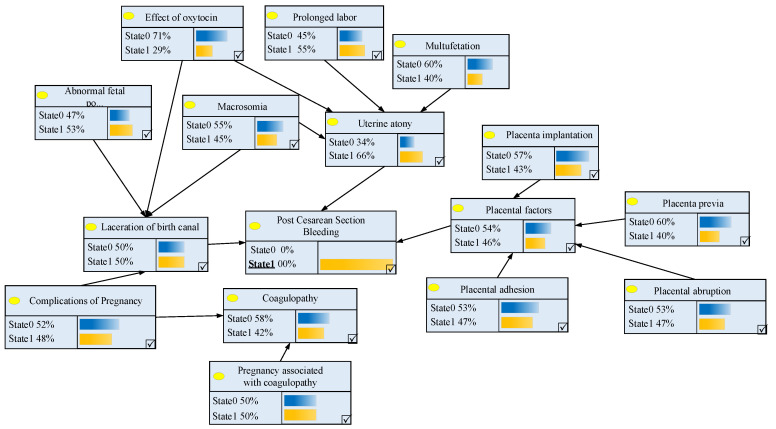
The posterior probability of bleeding risk factors after surgery.

In order to find out whether it is the primary risk factor or the secondary risk factor that has an effect on postpartum hemorrhage after cesarean section, we set the condition that postpartum hemorrhage has occurred in State 1, then calculate the posterior probability of each risk factor. The higher the posterior probability is, the more likely it will affect the likelihood of postpartum hemorrhage after cesarean section and confirm the key risk factors. The results of the calculation are sorted in Table 5 and illustrated in Figure 5. Table 5 shows that the most important primary risk factor is uterine atony, which has the largest probability of inducing postpartum hemorrhage.

On this basis, we can identify the risk factor with the highest posterior probability from the secondary risk factors that affect uterine atony, which is prolonged labor. A prolonged labor is not only the most severe factor affecting uterine atony, but also the most dangerous factor among all the secondary risk factors. It has the highest probability of occurrence in reality, hence severely affecting a gravida’s health.

### 3.2. Establishing the Response Mechanism of Postpartum Hemorrhage Variance

In order to prevent the occurrence of postpartum hemorrhage after cesarean section, the key factors leading to the occurrence of hemorrhage have been found through the Bayesian network. However, if there is an emergency situation of postpartum hemorrhage, it will be critical to understand how to formulate the corresponding hemorrhage treatment medical method according to the different causes and clinical characteristics of pregnant women. Association rules are a kind of data mining technology with a flexible feature that does not require a specific argument and function, and only needs to find the relationship among variables [29,30,31,32,33]. Using the technique of association rules mining, we can find the most appropriate medical treatment for postpartum hemorrhage.

In order to identify the association between postpartum hemorrhage and medical treatments, the Apriori algorithm of association rules mining was used to analyze the successful cases of handling postpartum hemorrhage after cesarean section [34,35]. On the basis of each risk factor analyzed in the last section, the risk factors appearing in the case of every gravida were regarded as clinical features, including the description of placental factors, whether there was uterine atony, whether the gravida had pregnancy complications, and so on. Meanwhile, specific medical treatments that were not used in the original data were regarded as treatments for postpartum hemorrhage, such as pharmaceuticals and operations. We deleted the data of incomplete records of processing methods. The clinical features, medication, and surgical treatment of pregnant women were selected as the research objects of association rule analysis. A total of 295 cases met the requirements of medical data, which covered a variety of clinical characteristics and medication treatment methods and provided a convincing basis for the experiment.

This following section mainly used the software Teradata Warehouse Miner to conduct data mining with association rules. The relationship between different risk factors, especially the combination of factors that are more likely to lead to postpartum hemorrhage in gravidas can be obtained in this chapter by rearranging the risk factors that caused postpartum hemorrhage for each gravida and analyzing them after rearrangement. The following is a brief description of the associated analysis results of double risk factors and triple risk factors for postpartum hemorrhage. Since the situation of quadruple or more risk factors occurring in one patient is so rare that it is unable to support association rule mining, it is not taken into consideration.

#### The Results of Multi-Factor Association Rules for Postpartum Hemorrhage

Due to the fact that a single risk factor is generally able to cause postpartum hemorrhage during cesarean section, it is less than normal for multiple factors to lead to morbidity. Because of this, the setting of the minimum threshold value is low. In order to obtain as many association rules as possible, experiments of multiple rule mining were used to determine the minimum value of each parameter. The minimum support for the double-factor analysis was 0.05, while the minimum confidence was 0.5 and the minimum lifting degree was 0.9. The situations that triple-factor appeared in the data were relatively scarce, so the minimum support degree was set to 0.12, and the other two values were the same. To order the degree of support from high to low, the rules that meet the conditions are laid out in Table 6 and Table 7.

In order to find the connection between postpartum hemorrhage and medical treatment, the algorithm in association rule mining, Apriori, was also used to analyze the successful treatment cases of postpartum hemorrhage after cesarean section. Given that methods for treating postpartum hemorrhage in the cases (which were recorded by different physicians) were slightly different in terms of the use of drugs, minimum support and confidence were set to a relatively low threshold to ensure regular generation on the basis of slightly changed descriptions to improve the specification of data representation and ensure the authenticity of data and feasibility of association rule analysis. The minimum degree of support, confidence, and lift in this section are 0.05, 0.15 and 1, respectively.

Based on the minimum degree of support and confidence, the above association rules algorithm is used via the software Teradata Warehouse Miner to study the association between cesarean section pathogeny and treatment methods. It was found that there are many valuable association rules, which were sorted by size of support (as shown in Table 8). Partial rules with a confidence of 1 are shown in Table 9.

By observing the association rules obtained in Table 8 and Table 9, the following conclusions can be drawn.

## 4. Discussion

### 4.1. The Meaning of the Prediction of an Individual Patient’s Risk of Variance

As is shown in Figure 4, the probability of postpartum hemorrhage is remarkably higher when gravidas have the specific symptoms listed above, which proves that the Bayesian network model can be used to predict the risk factors of individual patients. The risk prediction method presented in this paper can provide a means for medical providers to carry out risk assessment based on the different clinical features of pregnant women. Medical institutions could conduct a probability prediction of cesarean section for gravidas, while providing references for the clinical pathway processes and the timely responses of medical treatment methods.

### 4.2. The Significance of Identifying the Critical Risk Factors

To sum up, uterine atony is the leading cause of postpartum hemorrhage. Among the factors leading to uterine atony, prolonged labor is dominant. Analysis of key risk factors of postpartum hemorrhage after cesarean section is helpful for medical institutions to be able to take actions when confronted with certain situations, and to pay more attention to the causal factors behind these situations. Medical personnel who participate in clinical nursing of cesarean sections should pay more attention to the high-risk factors in the pathways mentioned in this paper so that they can improve clinical pathways. Further, this would allow them to observe the state of illness and prevent variance according to the constant physiological and psychological indexes that occur during the operational process. Controlling labor time can effectively reduce the occurrence of postpartum hemorrhage after cesarean section and improve the safety of gravidas. This paper provides some useful suggestions for medical providers in China by which to improve the medical service for cesarean section and prevent postpartum hemorrhage in advance.

### 4.3. The Application of Association Rules in Postpartum Hemorrhage Treatment

Through the analysis of Table 6 and Table 7, the following conclusions can be drawn intuitively.

Multi-factor rules with a higher degree of support all contain the factor uterine atony, and the confidence values of these rules are relatively high. Therefore, it can be seen from another point of view that uterine atony is the key factor causing postpartum hemorrhage.

The data in the table on postpartum hemorrhage of double-factor association rules indicate that if a gravida is in the situation where abnormal fetal position and uterine atony both occur during the surgery, the probability that she will have postpartum hemorrhage is greatest (the credibility of this rule is 80%). Therefore, the following advice is proposed for medical institutions. If abnormal fetal position is found in the preoperative examination, more attention should be paid to preventing the occurrence of uterine atony during surgery. Additionally, the amount of oxytocin and the length of labor time should be controlled strictly in case the gravida is in danger of postpartum hemorrhage.

Although the general degree of support for rules obtained through the analysis of triple-factor association rules was particularly low, it has a superior degree of confidence and promotion. The confidence was 1 in situations of postpartum hemorrhage where soft birth canal damage, abnormal fetal position, and uterine atony occurred at the same time. This indicates that when these three risk factors occur at the same time, the probability of sudden postpartum hemorrhage is 100%. The other two rules offer a similar conclusion, so if a gravida has three or more risk factors, her safety will be greatly threatened. If medical institutions have detected two of the risk factors before surgery, it is necessary to exclude the threats that can be treated and dealt with in advance, and thus reduce the risk of path change in gravidas.

Through the analysis of Table 8 and Table 9, the following conclusions can be derived.

(1) When gravidas show the phenomenon of uterine atony, the main treatment methods are anti-inflammatory treatment with cefotiam, anti-inflammatory oxytocin and fluid support therapy, and massaging the uterus and injecting carbetocin to enhance uterine contraction. The support degree of cefotiam treatment was the highest of the rules, indicating that it is mostly used when treating postpartum hemorrhage. Thus, it can be suggested that when gravidas are faced with That’s OK postpartum hemorrhage caused by uterine atony, giving cefotiam treatment is the best choice to promote their contraction capacity and inhibit the occurrence of postpartum hemorrhage.
(2) There are generally three postpartum hemorrhage treatment methods mentioned in the mined rules in Table 8: anti-inflammatory treatment with cefotiam, anti-inflammatory oxytocin, fluid support therapy, and massaging the uterus and injecting carbetocin. It has been proven that the main ways for big medical institutions to deal with postpartum hemorrhage after cesarean section are these three treatments. If gravidas have postpartum hemorrhage due to the combined effects of soft birth canal injury, abnormal fetal position, and uterine atony, the above three methods should be considered foremost to treat patients in time.
(3) In Table 9, a rule with a confidence rate of 1 represents the same solution used in the collection of data in medical institutions for the unification of certain clinical features. For example, when gravidas have postpartum hemorrhage because of anemia, uterine atony, or liver damage due to pregnancy, medical staff can employ ceftazidime for anti-inflammatory treatment. When gravidas have the problem of placental abruption, carboprost tromethamine injection is a feasible method for anti-inflammatory treatment. Because these rules have a high degree of confidence, it can be inferred that that they are the most effective solutions for treating postpartum hemorrhage caused by this pathogeny. This finding could be of great assistance to medical institutions in developing countermeasures for postpartum hemorrhage treatment.

## 5. Conclusions

Under the background of relatively weak management of variance, this paper used data mining methods to analyze postpartum hemorrhage after cesarean section with variance in the clinical pathway. Moreover, we established a Bayesian network model to analyze the risk factors of postpartum hemorrhage and by mining association rules we proposed corresponding solutions for postpartum hemorrhage resulting from different causes. The major conclusions are drawn as follows.

Firstly, by classifying different causes of postpartum hemorrhage after cesarean section, we divided the risk factors into primary and secondary risk factors. The four primary factors can directly affect the probability of postpartum hemorrhage after cesarean section, and the secondary factors can affect the primary factors. We next established the Bayesian network model corresponding to the risk factors, and calculated the probability of each node in the network. The probability of postpartum hemorrhage after cesarean section in general is 53%, implying that postpartum hemorrhage is a relatively high risk factor of clinical pathway variance. Further, postpartum hemorrhage is a main cause of death in gravidas during cesarean sections in China.

Secondly, by calculating the posterior probability of each node in the Bayesian network, we discovered the key factors that affect postpartum hemorrhage after a cesarean section. In this study, we sorted the posterior probability to obtain the key factors in the primary and secondary risk factors (i.e., uterine atony and prolonged labor) that put forward the most important monitoring indices when implementing the pathways for medical institutions.

Thirdly, the rules regarding clinical features associated with the management of postpartum hemorrhage during a cesarean section were obtained. This paper provides corresponding solutions to different causes of postpartum hemorrhage, and offers suggestions for medical institutions to improve the efficiency of variance treatment.

The data mining method used in this paper is only a preliminary attempt [36,37,38]. In addition, the cognition of the clinical pathway of cesarean sections is not complete. Therefore, it is hard to consider all aspects in the progression of the disease. In the future, some aspects need to be improved. First, the sample size of the clinical pathway collected in this paper could be increased, as obtaining more data samples and improving the accuracy of data mining analysis are important directions for future research. Second, this study only investigated single diseases that occur during cesarean section. In the future, data mining methods could be used to study more diseases and the variance handling of clinical pathways in various cases.

## Figures and Tables

**Table 1:** Risk factor system for postpartum hemorrhage.

Clinical Pathway Variance	Primary Risk Factors	Secondary Risk Factors
Postpartum hemorrhage in cesarean section	Uterine atony	Prolonged labor
		Multifetation
		Effect of oxytocin
		Macrosomia
	Coagulopathy	Pregnancy associated with coagulopathy Complications of pregnancy
	Placental factors	Placenta implantation
		Placenta previa
		Placental abruption
		Placental adhesion
	Laceration of birth canal	Effect of oxytocin
		Macrosomia
		Abnormal fetal position
		Complications of pregnancy

**Table 2:** Symbolic representation of nodes.

Symbols	Risk Factors
A	Postpartum hemorrhage in cesarean section
B_1_	Uterine atony
$B_{2}$	Laceration of birth canal
B_3_	Placental factors
$B_{4}$	Coagulopathy
C_1_	Prolonged labor
$C_{2}$	Multifetation
C_3_	Effect of oxytocin
$C_{4}$	Macrosomia
$C_{5}$	Abnormal fetal position
$C_{6}$	Complications of pregnancy
$C_{7}$	Placenta implantation
$C_{8}$	Placenta previa
$C_{9}$	Placental abruption
$C_{10}$	Placental adhesion
$C_{11}$	Pregnancy associated with coagulopathy

**Table 3:** Secondary risk factor probability of *B*_1_(uterine atony).

Secondary Risk Factor	Prolonged Labor	Multifetation	Effect of Oxytocin	Macrosomia
State 0	0.495	0.631	0.732	0.59
State 1	0.505	0.369	0.268	0.41

**Table 4:** Conditional probability of *B*_4_ (coagulopathy).

Pregnancy Associated with Coagulopathy	State 0		State 1	
Complications of Pregnancy	State 0	State 1	State 0	State 1
State 0	0.9	0.6	0.736	0.22
State 1	0.1	0.4	0.264	0.78

**Table 5:** Postpartum hemorrhage posterior probability calculation.

Symbol	Risk Factors	Posterior Probability (State 1)
$B_{1}$	Uterine atony	0.66
$B_{2}$	Laceration of birth canal	0.5
$B_{3}$	Placental factors	0.46
$B_{4}$	Coagulopathy	0.42
$C_{1}$	Prolonged labor	0.55
$C_{2}$	Multifetation	0.4
$C_{3}$	Effect of oxytocin	0.29
$C_{4}$	Macrosomia	0.45
$C_{5}$	Abnormal fetal position	0.53
$C_{6}$	Complications of pregnancy	0.48
$C_{7}$	Placenta implantation	0.43
$C_{8}$	Placenta previa	0.4
$C_{9}$	Placental abruption	0.47
$C_{10}$	Placental adhesion	0.47
$C_{11}$	Pregnancy associated with coagulopathy	0.5

**Table 6:** Postpartum hemorrhage of the double-factor association rules.

ID	Factor A	Factor B	Supp.	Conf.	Lift
1	Abnormal fetal position	Uterine atony	0.15	0.80	98
2	Soft birth canal damage	Uterine atony	0.13	0.95	1.15
3	Macrosomia	Uterine atony	0.10	0.73	0.92
4	Placental adhesion	Uterine atony	0.08	0.71	0.91
5	Multiple pregnancy	Uterine atony	0.06	0.79	0.96
6	Diabetes	Uterine atony	0.06	0.82	0.99
7	Hypertension	Uterine atony	0.05	0.89	1.08
8	Pelvic infection	Uterine atony	0.05	0.94	1.14

**Table 7:** Association rules table for the triple-factor analysis of postpartum hemorrhage.

ID	Factor A	Factor B	Factor C	Supp.	Conf.	Lift
1	Soft birth canal damage	Abnormal fetal position	Uterine atony	0.027	1	1.21
2	Multiple pregnancy	Abnormal fetal position	Uterine atony	0.017	1	1.21
3	Macrosomia	Soft birth canal damage	Uterine atony	0.013	1	1.21

**Table 8:** Association rules table of postpartum hemorrhage treatment methods.

ID	Clinical Features	Treatment	Supp.	Conf.	Lift
1	Uterine atony	Anti-inflammatory treatment of cefotiam	0.20	0.24	1.0
2	Uterine atony	Anti-inflammatory, oxytocin and fluid support therapy	0.14	0.17	1.11
3	Uterine atony	Massage the uterus and inject carbetocin	0.14	0.17	1.06
4	Soft birth canal damage	Anti-inflammatory treatment of cefotiam	0.08	0.61	2.51
5	Abnormal fetal position	Massage the uterus and to inject carbetocin	0.08	0.45	2.80
6	Soft birth canal damage, uterine atony	Anti-inflammatory treatment of cefotiam	0.076	0.59	2.43
7	Abnormal fetal position, uterine atony	Massage the uterus and to inject carbetocin	0.076	0.51	3.21
8	Diabetes	Anti-inflammatory treatment of cefotiam	0.053	0.73	3.00
9	Pelvic infection, uterine atony	Anti-inflammatory, oxytocin and fluid support therapy	0.053	0.87	5.55
10	Diabetes, uterine atony	Anti-inflammatory treatment of cefotiam	0.053	0.72	2.98

**Table 9:** Association rules table of postpartum hemorrhage treatment methods (Conf. = 1).

ID	Clinical Features	Treatment	Supp.	Conf.	Lift
1	Anemia, uterine atony	Anti-inflammatory treatment of cefotiam	0.033	1	8.6
2	Liver damage due to pregnancy, uterine atony	Anti-inflammatory treatment of cefotiam	0.027	1	8.5
3	Placental abruption	Carboprost Tromethamine injection treatment	0.023	1	37.6
3	Placental abruption	Carboprost Tromethamine injection treatment	0.023	1	37.6
